# Comparative Analysis of Gut Microbiota Between Healthy and Diarrheic Horses

**DOI:** 10.3389/fvets.2022.882423

**Published:** 2022-05-02

**Authors:** Yaonan Li, Yanfang Lan, Shuang Zhang, Xiaoli Wang

**Affiliations:** Wuhan Business University, Wuhan, China

**Keywords:** diarrhea, horse, dysbiosis, gut microbiota, healthy

## Abstract

Increasing evidence reveals the importance of gut microbiota in animals for regulating intestinal homeostasis, metabolism, and host health. The gut microbial community has been reported to be closely related to many diseases, but information regarding diarrheic influence on gut microbiota in horses remains scarce. This study investigated and compared gut microbial changes in horses during diarrhea. The results showed that the alpha diversity of gut microbiota in diarrheic horses decreased observably, accompanied by obvious shifts in taxonomic compositions. The dominant bacterial phyla (*Firmicutes, Bacteroidetes, Spirochaetes*, and *Kiritimatiellaeota*) and genera (*uncultured_bacterium_f_Lachnospiraceae, uncultured_bacterium_f_p-251-o5, Lachnospiraceae_AC2044_group*, and *Treponema_2*) in the healthy and diarrheic horses were same regardless of health status but different in abundances. Compared with the healthy horses, the relative abundances of *Planctomycetes, Tenericutes, Firmicutes, Patescibacteria*, and *Proteobacteria* in the diarrheic horses were observably decreased, whereas *Bacteroidetes, Verrucomicrobia*, and *Fibrobacteres* were dramatically increased. Moreover, diarrhea also resulted in a significant reduction in the proportions of 31 genera and a significant increase in the proportions of 14 genera. Taken together, this study demonstrated that the gut bacterial diversity and abundance of horses changed significantly during diarrhea. Additionally, these findings also demonstrated that the dysbiosis of gut microbiota may be an important driving factor of diarrhea in horses.

## Introduction

Mammal intestines contain more than 10^14^ microorganisms including bacteria, fungi, viruses, and protozoa, which are approximately 10 times the total quantity of host cells and play crucial roles in intestinal physiological function, metabolism, and host health (1–3). Furthermore, increasing evidence indicated that gut microbiota also functions in epithelial differentiation, intestinal homeostasis, and immunity (4, 5). Early investigations demonstrated that the consistency of the gut microbial community is the precondition for conducting digestive absorption and complicated metabolic functions, whereas gut microbial dysbiosis is closely related to many diseases (6–8). Currently, gut microbial dysbiosis has been shown to be an important driving factor of non-alcoholic fatty liver disease, high blood pressure, and diabetes (9, 10). Recent research on gut microbiota has also provided evidence that obesity, colonitis, and colorectal cancer may be the result of gut microbial dysbiosis (11, 12).

Diarrhea is one of the main reasons for decreased production performance and death in farmed animals and has been regarded as a key factor affecting the development of the livestock industry in many countries. Previous studies indicated that diarrhea was present in nearly all mammals, especially in newborn pigs, chickens, and sheep with susceptible gut microbiota (13–15). Considering the negative impact of diarrhea on animal husbandry, it is important to investigate its etiology and treatment. Numerous studies indicated that gut microbiota played key roles in the prevention, control, and diagnosis of diarrhea (16, 17). Wang et al. revealed that the gut microbial community of diarrheic goats changed dramatically accompanied by high mortality (13). Similarly, Li et al. also reported that the gut microbiota of giraffes changed significantly during diarrhea (18).

Metagenomics is a key tool for investigating shifts in gut microbiota during diseases (19, 20). By systematically exploring and comparing acquired information, the relationship between gut microbiota and diseases could be further understood, and prevention and control measures can be developed to minimize economic losses (21–23). Presently, the complicated composition and structure of gut microbiota in diarrheic pigs, yaks, and giraffes have been successfully analyzed based on the high-throughput sequencing technology (24–26). However, there are few reports on the gut microbiota of horses, and even fewer studies on the composition and structure of gut microbiota in horses in different health statuses. Therefore, the objective of this study was to compare and investigate the composition and discrepancy of gut microbial populations between healthy and diarrheic horses.

## Materials and Methods

### Animals and Sample Collection

A total of 16 horses (8 healthy horses and 8 diarrheic horses) from Wuhan Business University (Wuhan, China) were used for this experiment. The horses we screened possessed the same immune background. Moreover, the health statuses of the horses were diagnosed and evaluated by a professional veterinarian before sample collection. The rectum was swabbed by a trained technician using sterile swabs in a rotating fashion. The obtained samples including healthy and diarrheic feces were immediately placed into sterile plastic containers and transported to the laboratory and later stored at −80°C for further study.

### 16S rDNA Gene Amplicon Sequencing

Prior to the DNA extraction, 16 fecal samples from control and diarrheic horses were unfrozen and homogenized at room temperature. Afterward, the treated fecal samples were subjected to bacterial DNA extraction based on the manufacturer's protocol. Quantification and electrophoresis of the extracted DNA were performed to ensure that the concentration and integrity of extracts meet analysis demands. To dissect the changes in the gut bacterial community, we amplified the V3/V4 regions utilizing bacterial primers (338F: ACTCCTACGGGAGGCAGCA and 806R: GGACTACHVGGGTWTCTAAT). The PCR amplification procedure was set based on previous studies. PCR products were conducted target fragment recovery and gel electrophoresis detection to acquire purified products. The PCR products were recovered by fluorescence quantification and proportionally mixed following sequencing requirements. The qualified products were used to prepare sequencing libraries by using the PacBio platform (Biomarker Technologies, China). To acquire qualified libraries, the original libraries were required to suitably embellish such as sequence repair, quality evaluation, purification, and fluorescent quantitation. Libraries that passed quality screening were subjected to 2 × 300 bp paired-end sequencing using a MiSeq sequencing machine.

### Bioinformatics and Data Analysis

The initial data from Illumina MiSeq sequencing was performed a quality assessment to obtain effective data. Briefly, raw data containing problematical sequences including short, unqualified, and mismatched sequences were subjected to screening and removal of primer sequences to achieve clean reads utilizing the Trimmomatic (v0.33) and Cutadapt software (1.9.1). The Usearch software (v10) was used for splicing clean reads and then the spliced sequences were secondary screened based on sequence length range. Subsequently, identification and elimination of chimera sequences were performed to obtain final effective reads utilizing the UCHIME software (v4.2). Effective reads that passed quality inspection were clustered, and OTUs were partitioned based on 97% similarity. Additionally, Venn maps were also generated to characterize the distribution and richness of bacterial OTUs in each sample. To further investigate the shifts in gut microbial diversity and abundance during diarrhea, we computed multiple alpha diversity indexes based on OTU distribution. Principal component analysis was also conducted to dissect gut bacterial beta diversities between both groups. The sequencing depth and evenness of each sample were evaluated through rank abundance and rarefaction curves. Differential bacterial taxa associated with diarrhea exposure were recognized by Metastats and LEfSe analysis. An SPSS statistical program (v20.0) was used for conducting data analysis, and *P*-values (means ± SD) <0.05 were determined statistically significant.

## Results

### Sequences Analyses

**Figure 1:**
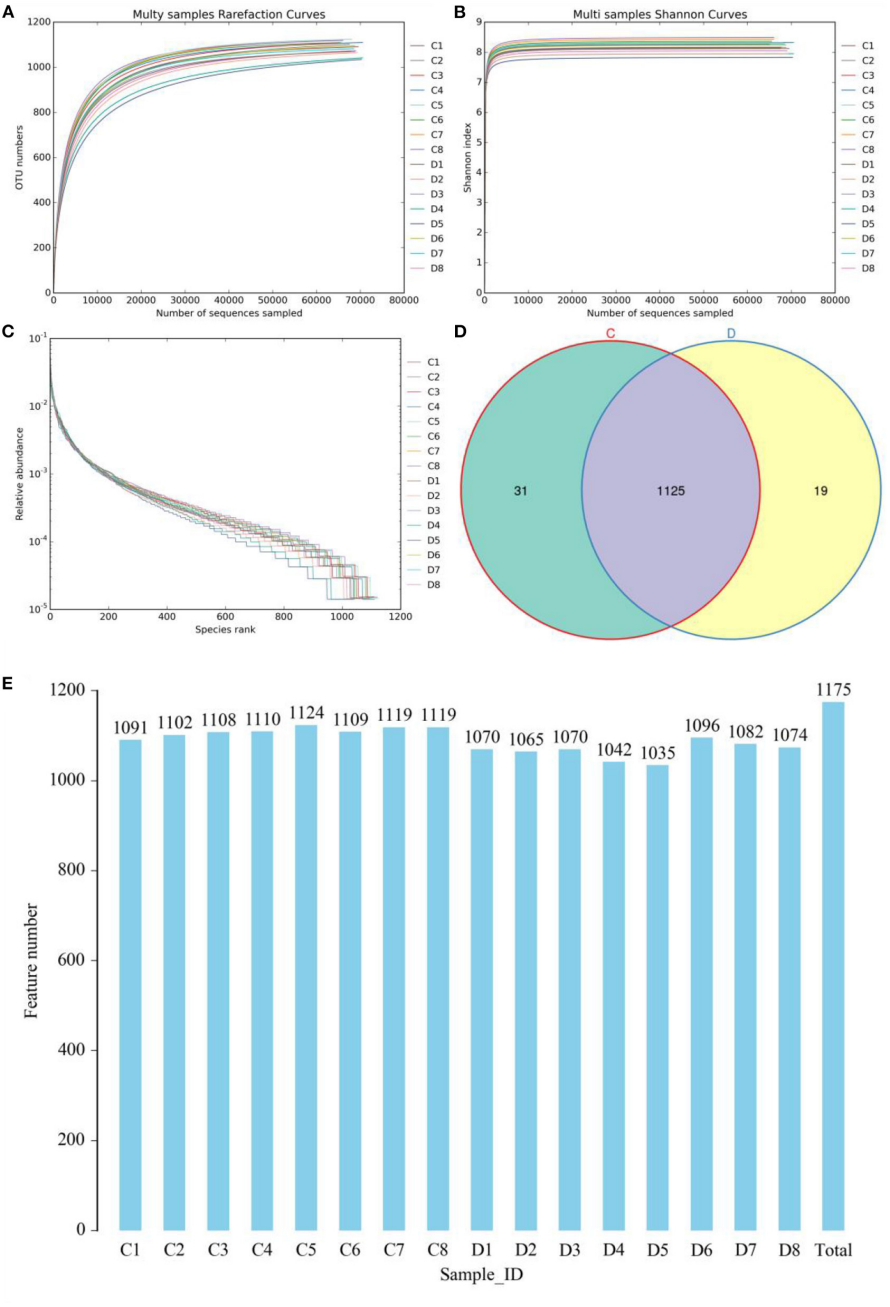
Feasibility analysis of sequencing data. Sequencing depth and evenness of gut microbiota could be assessed with **(A,B)** rarefaction and **(C)** rank abundance curves. **(D)** Venn diagrams for shared and unique operational taxonomic unit (OTU) distribution. **(E)** Quantity of OTUs in each sample.

In this research, 8 healthy and 8 diarrheic fecal samples were subjected to high-throughput sequencing analysis. After optimizing the original data, a total of 127,8741 high-quality sequences were obtained from the 16 samples (Table 1). In addition, the number of valid sequences in the healthy horses ranged from 794.84 to 803.09, while the number of valid sequences in the diarrheic populations varied from 794.85 to 801.39. The Chao1, Shannon, and Rank abundance curves showed a tendency to saturate, implying eligible depth and evenness (Figures 1A–C). High-quality sequences with 97% nucleotide sequence similarity were identified as one OTU. A total of 1,175 OTUs have been recognized in gut bacterial communities, varying from 1,035 to 1,124 in each sample (Figure 1E). Moreover, there were 1,156 and 1,144 OTUs in the healthy and diarrheic horses, respectively, and 1,125 OTUs in common, accounting for approximately 95.74% of the total OTUs (Figure 1D).

**Table 1:** Bacterial sequence information of each sample.

**Sample**	**Raw reads**	**Clean reads**	**Effective reads**	**AvgLen (bp)**	**GC (%)**	**Q20 (%)**	**Q30 (%)**	**Effective (%)**
CH1 CH2 CH3 CH4 CH5 CH6 CH7 CH8 DH1 DH2 DH3 DH4 DH5 DH6 DH7 DH8	79824 79487 79834 79951 79990 80309 79670 79921 79485 80084 79807 80152 79940 80139 80081 80067	79497 79172 79480 79625 79678 79962 79355 79622 79167 79777 79505 79833 79603 79838 79776 79774	78225 77656 77935 78834 78425 77878 77824 78072 77895 78397 78279 78676 78517 78773 78666 78525	413 415 414 413 413 415 414 414 414 414 414 414 414 416 415 413	52.74 52.69 52.68 52.88 52.71 52.68 52.81 52.91 53.10 52.88 53.00 52.97 52.69 52.90 52.94 53.02	99.07 99.06 99.06 99.09 99.06 99.08 99.07 99.09 99.06 99.06 99.05 99.08 99.03 99.05 99.04 99.08	96.12 96.09 96.09 96.18 96.10 96.14 96.12 96.17 96.09 96.10 96.08 96.16 96.02 96.06 96.04 96.15	98.00 97.70 97.62 98.60 98.04 96.97 97.68 97.69 98.00 97.89 98.09 98.16 98.22 98.30 98.23 98.07

### Analysis of Microbial Diversity in the Healthy and Diarrheic Horses

**Figure 2:**
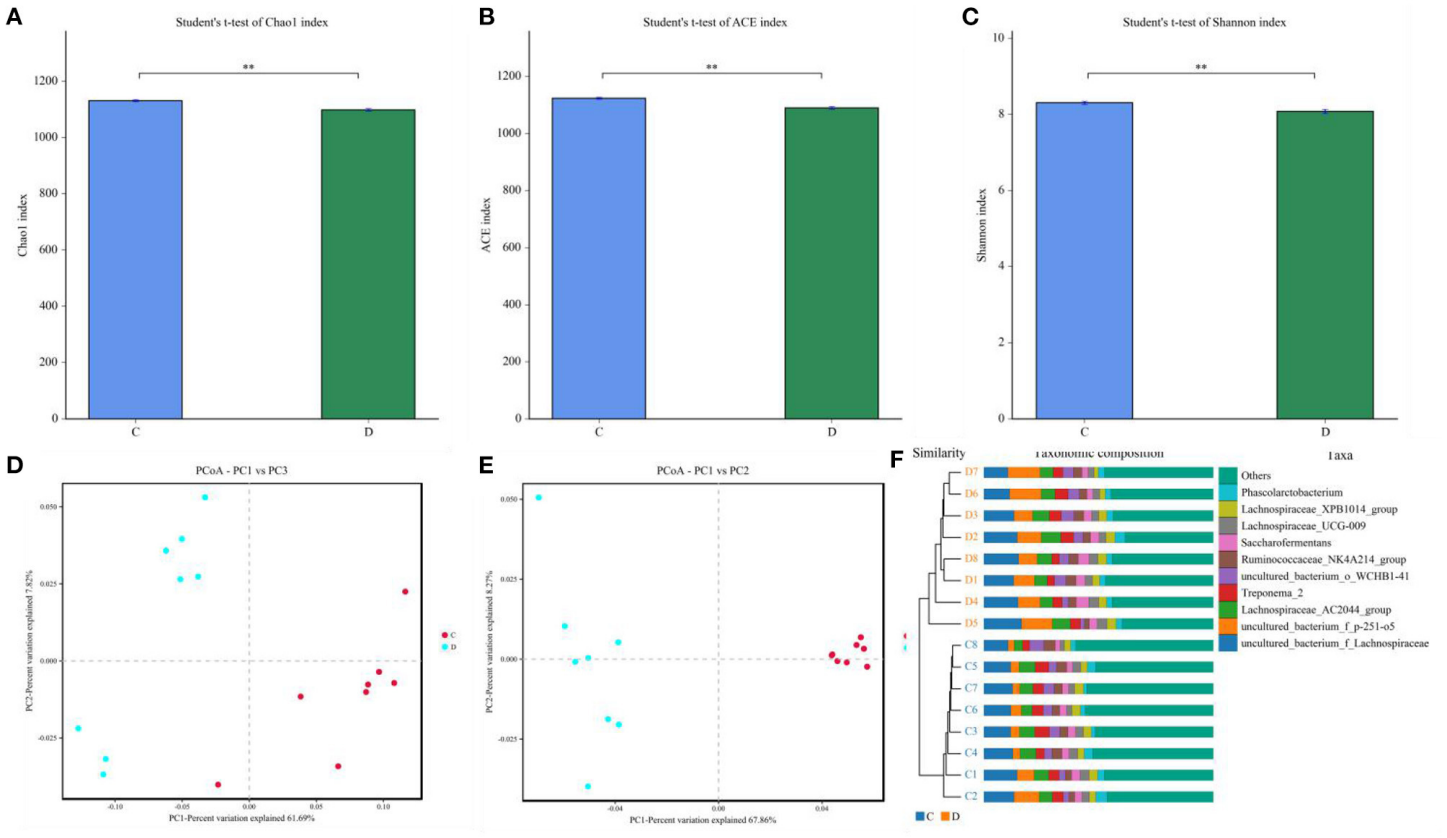
Comparative analysis of alpha and beta diversity of gut microbiota between the healthy and diarrheic horses. **(A–C)** represent Chao, ACE, and Shannon indices, respectively. **(D,F)** indicate PCoA map based on **(E)** weighted and **(F)** unweighted UniFrac distance. **(F)** Clustering analysis map.

The indicates of Chao1, ACE, Shannon, and Good's coverage were calculated to evaluate the alpha diversity of the microbial community. Good's coverage estimates varied from 99.88 to 99.96% for all of the samples, showing excellent coverage. The average Chao1 and ACE indices in the healthy horses were 1,130.99 and 1,123.46, while those in the diarrheic populations were 1,098.34 and 1,089.36 (Figures 2A,B). Furthermore, the average Shannon index was 5.861 and 6.41 in the healthy and diarrheic horses, respectively (Figure 2C). Statistical analysis showed that the diversity indices including Chao1, ACE, and Shannon of the healthy horses were significantly higher than those of the diarrheic populations. The results of Chao1, ACE, and Shannon indices showed that there were significant differences in the richness and diversity of gut microbial population between the healthy and diarrheic horses. The PCoA scatterplot of gut microbiota showed a separation of samples in the healthy and diarrheic horses, which was in line with the UPGMA results, indicating a significant shift in gut microbial principal compositions (Figures 2D–F).

### Composition Analysis of the Gut Microbial Community in the Healthy and Diarrheic Horses

**Figure 3:**
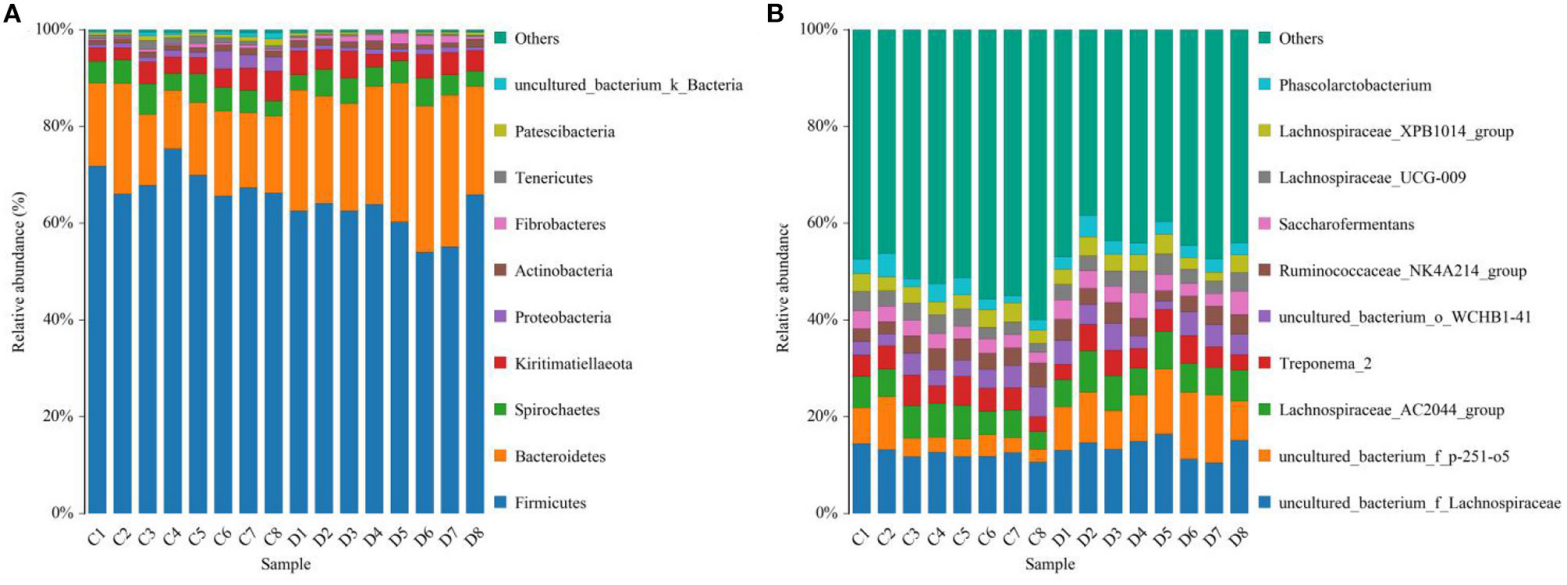
Proportion of preponderant bacterial **(A)** phyla and **(B)** genera in the healthy and diarrheic horses.

**Figure 4:**
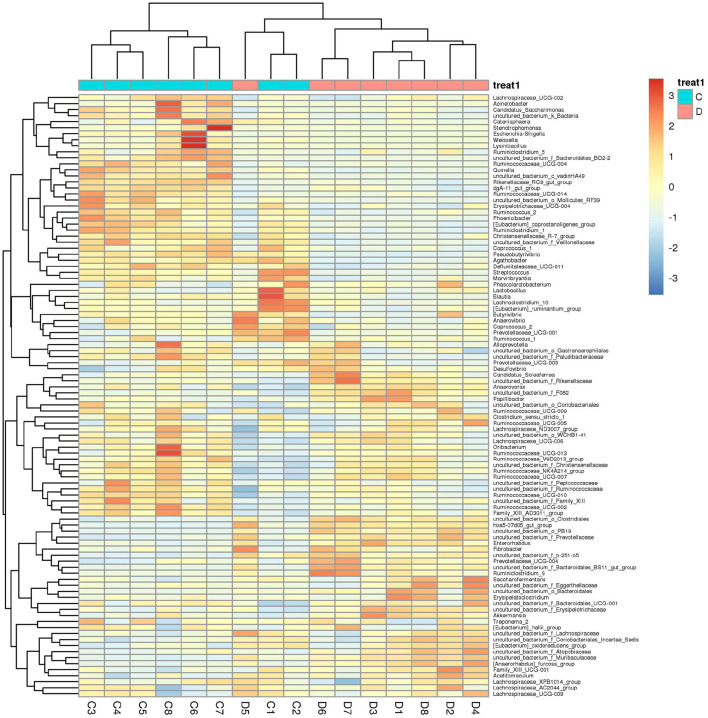
Heatmap of the genus-level hierarchical clustering of the microbial community in the healthy and diarrheic horses.

Gut microbial community composition in the healthy and diarrheic horses was assessed at different taxonomical levels. At the phylum level, *Firmicutes* (61.07, 68.87%), *Bacteroidetes* (25.77, 16.29%), *Spirochaetes* (4.48, 4.72%), and *Kiritimatiellaeota* (4.01, 3.88%) were dominant in the healthy and diarrheic horses regardless of health statuses (Figure 3A). Moreover, other phyla such as *Actinobacteria* (1.23, 1.1%), *Fibrobacteres* (1.25, 0.47%),*Tenericutes* (0.33, 1.05%), and *Patescibacteria* (0.4, 0.75%) in both groups were represented with a lower abundance. At the level of genus, *uncultured_bacterium_f_Lachnospiraceae* (13.67, 12.36%), *uncultured_bacterium_f_p-251-o5* (10.79, 4.94%), *Lachnospiraceae_AC2044_group* (6.61, 5.88%), and *Treponema_2* (4.47, 4.7%) were the predominant bacteria in both groups (Figure 3B). The heatmap also displayed the distribution and variability of the bacterial genera in the diarrheic horses (Figure 4).

**Figure 5:**
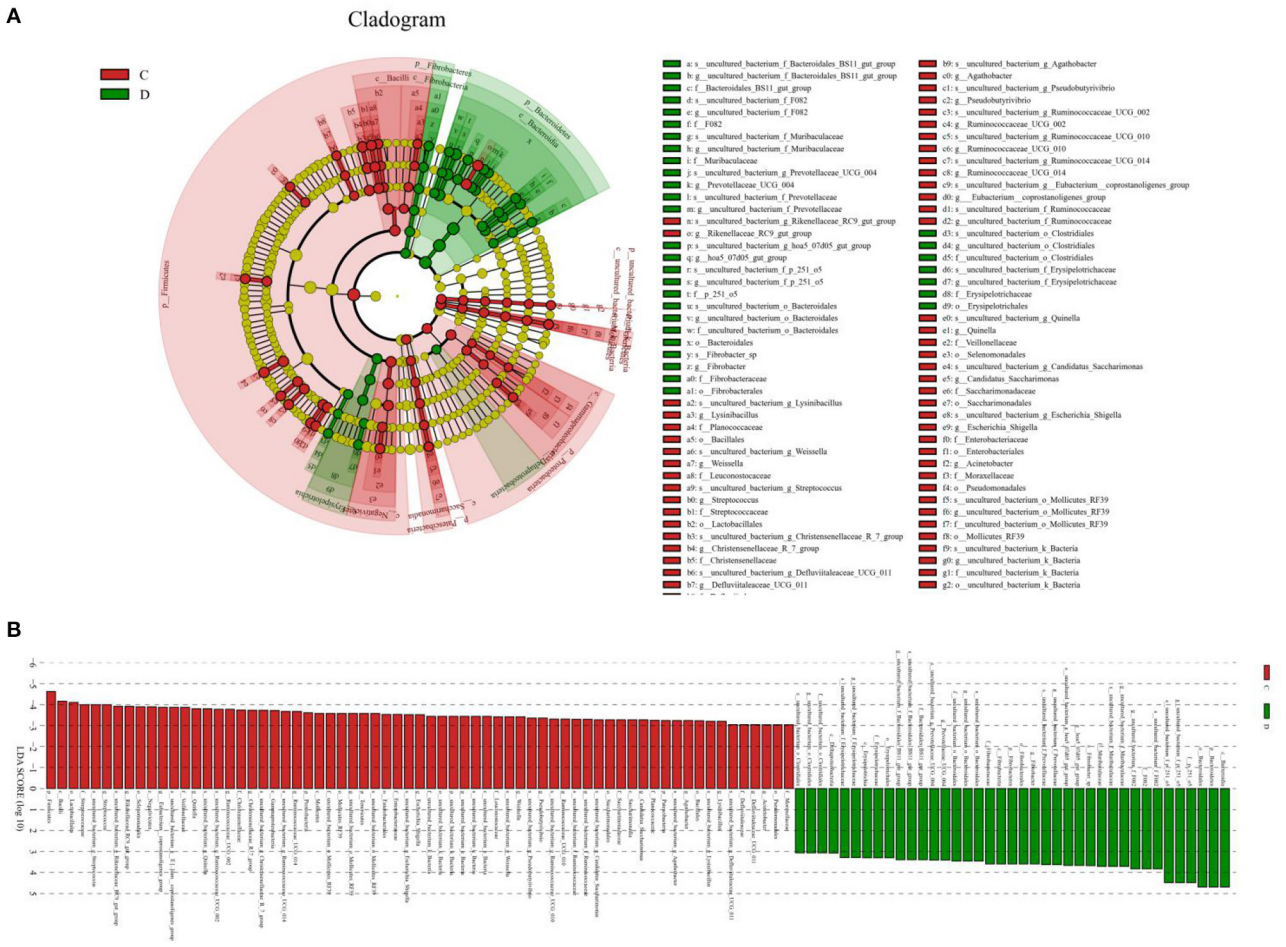
Differential biomarkers in gut microbiota of the horses associated with diarrhea. **(A)** Phylogenetic distribution of taxa with significant differences are visualized through the cladogram. **(B)** The criterion of significance was determined at LDA scores > 3.

A comparison of gut microbiota at the levels of phylum and genus was also conducted between the healthy and diarrheic horses. At the level of phylum, the relative abundances of *Bacteroidetes, Fibrobacteres*, and *Verrucomicrobia* in the diarrheic horses were significantly higher than in the healthy populations, while the *Planctomycetes, Tenericutes, Firmicutes, Patescibacteria*, and *Proteobacteria* contents were lower (Table 2). Moreover, a comparison of the diarrheic and healthy horses showed a significant increase in the abundance of 14 genera (*Breznakia, Enterorhabdus, Mailhella, Oscillospira, Proteus, Anaerorhabdus_furcosa_group, Erysipelotrichaceae_UCG-009, Prevotellaceae_UCG-004, Fibrobacter, Parvibacter, Acetitomaculum, Pygmaiobacter, Succinivibrionaceae_UCG-002*, and*Candidatus_Soleaferrea*) as well as an obvious reduction in the abundance of 31 genera (*Anaerofustis, Christensenellaceae_R-7_group, Defluviitaleaceae_UCG-011, Glutamicibacter, Lysinibacillus, Phoenicibacter, Pseudobutyrivibrio, Quinella, Ruminiclostridium_1, Ruminiclostridium_6, Ruminococcus_2, Selenomonas_1, Shuttleworthia, Solibacillus, Weissella, Eubacterium_coprostanoligenes_group, Marvinbryantia, Rikenellaceae_RC9_gut_group, Erysipelotrichaceae_UCG-004, Blautia, Candidatus_Saccharimonas, Coprococcus_1, Ruminococcaceae_UCG-014, Agathobacter, Ruminococcaceae_UCG-004, Vagococcus, Kurthia, Lachnospiraceae_UCG-002, Eubacterium_ruminantium_group, Brevibacterium*, and *Ruminococcaceae_UCG-002*). LEfSe combined with LDA scores was conducted to further dissect the shifts in gut microbiota. Besides the above-mentioned differential taxa, the diarrheic horses also showed significantly higher abundances of *Acinetobacter, Ruminococcaceae_UCG_010*, and *Streptococcus*, and low abundances of *Fibrobacter* (Figures 5A,B).

**Table 2:** Statistical comparison of differential taxa between the healthy and diarrheic horses. All the data are represented as mean ± SD.

**Taxa**	**C (%)**	**D (%)**	**P**
Bacteroidetes Planctomycetes Tenericutes Firmicutes Fibrobacteres Patescibacteria Verrucomicrobia Proteobacteria Anaerofustis Breznakia Christensenellaceae_R-7_group Defluviitaleaceae_UCG-011 Enterorhabdus Glutamicibacter Lysinibacillus Mailhella Oscillospira Phoenicibacter Proteus Pseudobutyrivibrio Quinella Ruminiclostridium_1 Ruminiclostridium_6 Ruminococcus_2 Selenomonas_1 Shuttleworthia Solibacillus Weissella [Anaerorhabdus]_furcosa_group [Eubacterium]_coprostanoligenes_group Erysipelotrichaceae_UCG-009 Marvinbryantia Prevotellaceae_UCG-004 Rikenellaceae_RC9_gut_group Erysipelotrichaceae_UCG-004 Blautia Candidatus_Saccharimonas Coprococcus_1 Fibrobacter Ruminococcaceae_UCG-014 Agathobacter Parvibacter Ruminococcaceae_UCG-004 Vagococcus Acetitomaculum Kurthia Pygmaiobacter Lachnospiraceae_UCG-002 Succinivibrionaceae_UCG-002 [Eubacterium]_ruminantium_group Brevibacterium Ruminococcaceae_UCG-002 Solobacterium Candidatus_Soleaferrea	16.3 0.0851 1.05 68.8 0.476 0.755 0.089 1.81 0.0355 0.000905 2.82 0.521 0.0592 0.0205 0.34 0.00569 0.000185 0.0939 0.00351 1.06 1.72 0.071 0.0226 0.0909 0.0262 0.012 0.0429 0.577 0.0541 2.76 0.00628 0.118 0.824 2.92 0.205 0.282 0.755 0.0538 0.476 1.62 0.882 0.0132 0.216 0.0176 0.0342 0.0151 0.0105 0.142 0.00186 0.164 0.0156 3.01 0.00732 0.185	25.8 0.00525 0.337 61.1 1.24 0.397 0.174 0.917 0.000181 0.0116 1.74 0.316 0.126 0.00451 0.00761 0.0435 0.0134 0.0219 0.0166 0.606 0.425 0.0154 0.00235 0.0406 0.00451 0.000903 0.00688 0.0101 0.18 1.35 0.0152 0.0725 1.3 1.25 0.112 0.206 0.371 0.0311 1.24 0.704 0.531 0.0252 0.0861 0.000718 0.0524 0.000544 0.0349 0.0852 0.00952 0.117 0.00233 1.85 0.0161 0.356	0.000999 0.000999 0.000999 0.002 0.004 0.005 0.014 0.039 0.000999 0.000999 0.000999 0.000999 0.000999 0.000999 0.000999 0.000999 0.000999 0.000999 0.000999 0.000999 0.000999 0.000999 0.000999 0.000999 0.000999 0.000999 0.000999 0.000999 0.000999 0.000999 0.002 0.002 0.002 0.002 0.003 0.004 0.004 0.004 0.004 0.004 0.00599 0.00899 0.00899 0.011 0.012 0.014 0.015 0.016 0.016 0.017 0.018 0.02 0.028 0.04

### Correlation Network Analysis

**Figure 6:**
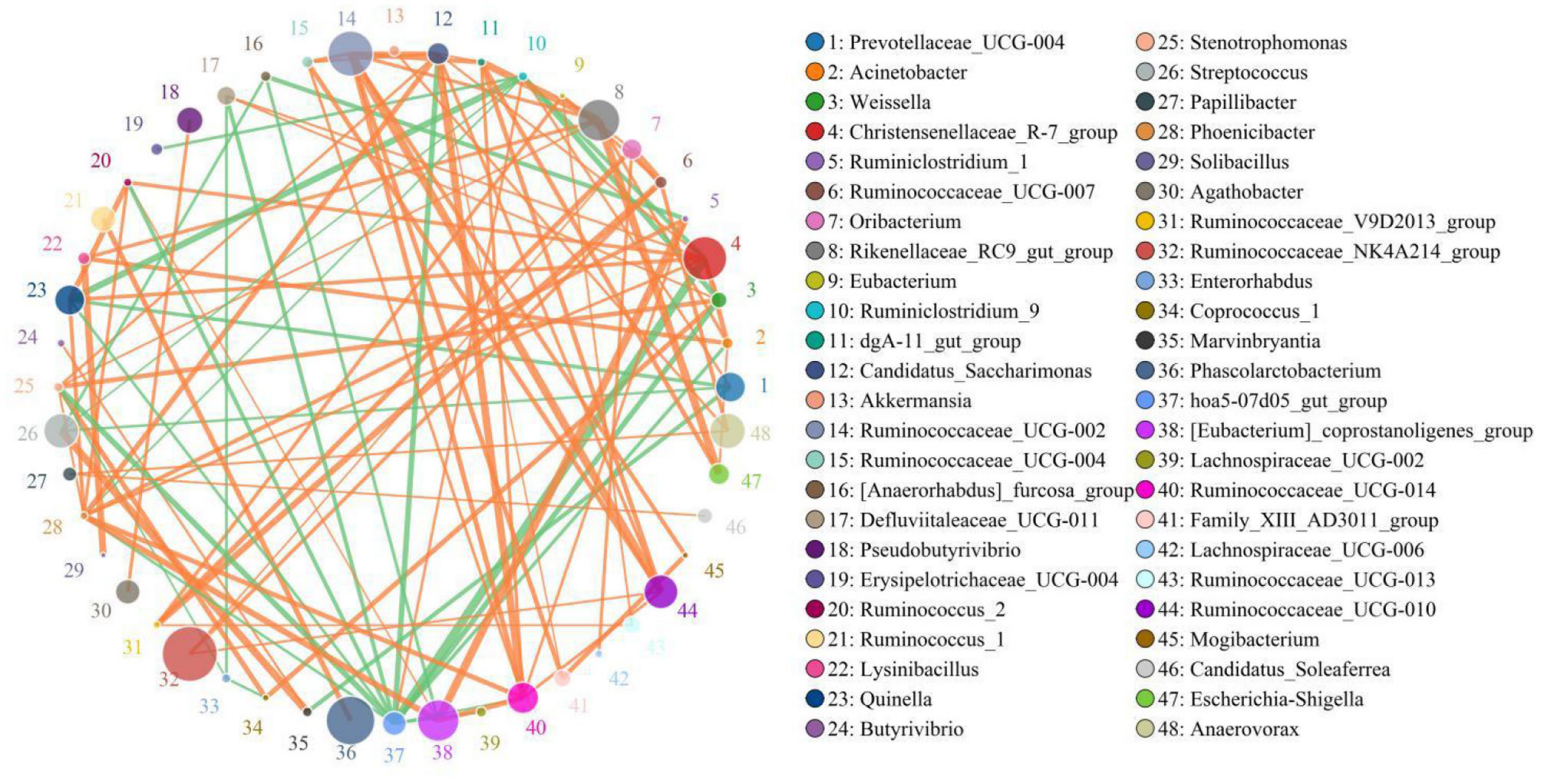
Network analysis indicates a connection among different bacteria. The orange lines indicate positive correlation and the green lines indicate negative correlation.

The results indicated that *Christensenellaceae_R-7_group* was positively associated with *Weissella* (0.8206), *Phoenicibacter* (0.8206), *Quinella* 0.8412), *Defluviitaleaceae_UCG-011* (0.8088), and *Ruminococcus_2* (0.8382) (Figure 6). *Defluviitaleaceae_UCG-011* was positively correlated with *Weissella* (0.8059). *Rikenellaceae_RC9_gut_group* was positively related to *Lysinibacillus* (0.8344) and *Stenotrophomonas* (0.803). *Ruminococcaceae_UCG-002* was positively correlated with *Ruminococcaceae_UCG-014* (0.8647), *Ruminococcaceae_UCG-010* (0.9324), and *Mogibacterium* (0.8471). *Ruminococcaceae_UCG-014* was positively associated with *Phoenicibacter* (0.8618), *Ruminococcaceae_UCG-004* (0.8206), and *Ruminiclostridium_1* (0.8176). *Weissella* was positively correlated with *Coprococcus_1* (0.8647) and *Stenotrophomonas* (0.8608).

## Discussion

Gut microbiota are a complicated and interactive ecosystem involving trillions of microbes (27, 28). Gut microbial interaction plays a key role in host health (29, 30). Gut microbiota could decrease the invasion and colonization of pathogens by regulating the intestinal barrier and environment, indicating its vital role in gastrointestinal diseases (31, 32). Consequently, the investigation of gut microbiota has attracted widespread attention. However, only a few studies have been conducted to investigate the gut microbiota in horses with different health statuses. In this study, we compared and analyzed gut microbial differences between healthy and diarrheic horses.

Gut microbial diversity and abundance change dynamically within certain limits and affect by age, diet, and environment, but these normal changes cannot damage intestinal functions (33, 34). However, some intense stimuli and diseases such as heavy metals, antibiotics, and gastrointestinal diseases may destroy ecological balance and induce gut microbial dysbiosis (35–37). Li et al. revealed reduced alpha diversity of gut microbiota in giraffes during diarrhea (18). Furthermore, He et al. reported that the gut microbial diversity of piglets with diarrhea was significantly decreased (38). In this study, we observed that diarrhea results in a significant reduction in gut microbial diversity of horses, indicating gut microbial dysbiosis. Research showed that higher gut microbial abundance and diversity were conducive to maintaining intestinal homeostasis and functions (39). Conversely, gut microbial dysbiosis may impair intestinal barrier functions and mucosal immunity, which, in turn, increases morbidity caused by pathogenic bacteria and opportunistic pathogens (40, 41). Consequently, diarrheic horses suffering from gut microbial dysbiosis are at increased risk of bowel dysfunction and other diseases. A PCoA was conducted to dissect the effect of diarrhea on gut microbial main components of horses. The results demonstrated that the samples of healthy horses were clustered together and separated from the diarrheic samples, suggesting that the main components of gut microbiota changed significantly under the influence of diarrhea. Although all the selected horses possessed the same diet and environment, the gut microbiota changed during diarrhea. Consequently, we suspected that diarrhea was the main driving force of gut microbial dysbiosis in horses.

This research indicated that *Firmicutes* and *Bacteroidetes* were the most preponderant bacterial phyla in horses regardless of health status, which was consistent with previous findings on other mammals such as pigs, cattle, and goats, implying their key roles in intestinal ecology and function (42, 43). However, although the species of the dominant phyla were not altered, their abundances changed dramatically. In this study, we observed that the proportions of *Bacteroidetes, Fibrobacteres*, and *Verrucomicrobia* in the gut microbial community of the horses were significantly increased during diarrhea. Interestingly, Li et al. also reported that these bacterial phyla in the gut microbiota of diarrheic giraffes were significantly increased (18). For herbivores, *Firmicutes* participated in the degradation of cellulose, which is essential for nutrition and energy intake (44). Moreover, most members of *Firmicutes* are regarded as intestinal beneficial bacteria, showing positive regulation of intestinal homeostasis, disease resistance, and growth performance (45, 46). *Proteobacteria* exhibits multiple metabolic functions that contribute to meeting host nutrient and energy requirements (44).

We also found significant changes in some bacterial genera during diarrhea, which may play key roles in gut microbial balance and the development of diarrhea. Moreover, some dramatically decreased bacterial genera in the diarrheic horses including *Ruminiclostridium, Ruminococcus, Rikenellaceae, Christensenellaceae, Pseudobutyrivibrio, Weissella, Eubacterium*_*coprostanoligenes, Ruminococcaceae, Lachnospiraceae, Blautia, Lachnospiraceae*, and *Coprococcus*and *Blautia* are considered as intestinal beneficial bacteria and are critical for intestinal functions and host health. *Ruminiclostridium*, which mostly resides in the gastrointestinal tract, displayed the characteristics of decreasing gastrointestinal diseases and improving the growth performance of animals (47). Previous studies have reported that *Ruminococcus* participated in the degradation of starch and cellulose (48). *Rikenellaceae* has been previously demonstrated to degrade plant-derived polysaccharides as well as control colitis by stimulating the differentiation of T-regulatory cells (49). As a recognized beneficial bacterium, *Christensenellaceae* not only is associated with immunoregulation and host health but also contributes to the regulation of intestinal homeostasis and the environment (50). Moreover, *Christensenellaceae* can also produce several hydrolases including β-glucosidase, β-galactosidase, and α-arabinosidase (24). *Pseudobutyrivibrio* can produce butyrate, which is conducive to reducing angiocardiopathy and diabetes by activating brown adipose tissues (51). Moreover, recent investigations on butyrate-producing bacteria have provided evidence that they were potentially intestinal beneficial bacteria because of their important roles in alleviating inflammatory bowel disease and regulating immunologic functions (52, 53). *Weissella* exhibits the characteristics of antioxidation and anti-inflammatory, which contributes to maintaining intestinal homeostasis and improving disease resistance of the host (54). Additionally, *Weissella* has been reported to reduce fat accumulation and protect the liver in mice induced by a high-fat diet (55). Earlier research indicated that the relative abundance of *Eubacterium_coprostanoligenes* in the intestine was negatively correlated to the severity of anxiety (56). *Eubacterium_coprostanoligenes* also displayed the characteristics of reducing cholesterol (57). Numerous bodies of evidence demonstrated that *Ruminococcaceae* was primarily responsible for digesting starch and cellulose and showed positive regulation of intestinal homeostasis and environment (58). Notably, the higher abundance of *Ruminococcaceae* contributes to reducing intestinal permeability, non-alcoholic fatty liver, and liver cirrhosis (59, 60). *Lachnospiraceae* plays an important role in intestinal homeostasis by ameliorating intestinal inflammation (61). Remarkably, some of the above-mentioned bacteria such as *Blautia, Lachnospiraceae, Coprococcus, Ruminococcaceae, Ruminococcus*, and *Ruminiclostridium* were considered producers of short-chain fatty acids (SCFAs) (47). Consistent with this study, several previous research studies on other animals have also indicated a significant reduction in SCFA-producing bacteria during diarrhea (18, 62). Previous studies have indicated that SCFAs not only participated in the positive regulation of intestinal homeostasis, immunization, and barrier function but also play key roles in reducing inflammation and regulating energy intake (63, 64). Importantly, SCFAs can also inhibit the proliferation of pathogenic bacteria, showing significant effects of improving the intestinal environment to prevent diseases (65). These decreased beneficial bacteria in diarrheic horses play key roles in maintaining host health and intestinal homeostasis. Consequently, we speculated that these decreased bacteria may be important drivers of diarrhea in horses. Notably, we also observed that some decreased intestinal beneficial bacteria showed a significant correlation with other bacteria. It suggested that diarrhea can also indirectly impair other bacteria by interaction, which may further enhance the influence of diarrhea on the gut microbial community and induce gut microbial dysbiosis.

In summary, this study first explored changes in the gut microbiota in diarrheic horses. The results showed that diarrhea dramatically decreased the gut microbial diversity and altered the taxonomic composition, characterized by a reduced percentage of intestinal beneficial bacteria. This study fills in the gaps in the characteristics of gut microbiota in healthy and diarrheic horses and conveys a vital message that gut microbial dysbiosis may be one of the causes of diarrhea in horses. Importantly, this study contributes to the prevention and treatment of diarrheic horses from the gut microbial perspective.

## Data Availability Statement

The datasets presented in this study can be found in online repositories. The names of the repository/repositories and accession number(s) can be found below: https://www.ncbi.nlm.nih.gov/, PRJNA808959.

## Ethics Statement

The animal study was reviewed and approved by the Ethics Committee of the Wuhan Business University.

## Author Contributions

YanL and YaoL conceived and designed the experiments. YaoL contributed to sample collection and preparation. YaoL analyzed the data. YaoL wrote the manuscript. SZ and XW revised the manuscript. All authors reviewed the manuscript. All authors contributed to the article and approved the submitted version.

## Funding

The study was supported by the Wuhan Business University project (No. 2019KY003).

## Conflict of Interest

The authors declare that the research was conducted in the absence of any commercial or financial relationships that could be construed as a potential conflict of interest.

## Publisher's Note

All claims expressed in this article are solely those of the authors and do not necessarily represent those of their affiliated organizations, or those of the publisher, the editors and the reviewers. Any product that may be evaluated in this article, or claim that may be made by its manufacturer, is not guaranteed or endorsed by the publisher.
